# Effectiveness of ensitrelvir for cough caused by COVID-19 Omicron variant in patients with asthma

**DOI:** 10.1128/spectrum.03407-24

**Published:** 2025-04-15

**Authors:** Naoyuki Miyashita, Nobuyuki Horita, Yasushi Nakamori, Makoto Ogata, Naoki Fukuda, Akihisa Yamura, Tomoki Ito

**Affiliations:** 1 First Department of Internal Medicine, Division of Respiratory Medicine, Infectious Disease and Allergology, Kansai Medical University 12880, Hirakata, Japan; 2 Chemotherapy Center, Yokohama City University Hospital 218758, Yokohama, Japan; 3 Department of Emergency Medicine, Kansai Medical University Medical Center, Hirakata, Japan; MultiCare Health System, Tacoma, Washington, USA

**Keywords:** cough symptom, ensitrelvir, Omicron variant, COVID-19, SARS-CoV-2

## Abstract

In the post-acute coronavirus disease 2019 (COVID-19) period, patients with asthma had a significantly higher risk of cough than patients without a history of asthma. In particular, cases with persistent cough were increased during the Omicron variant epidemic. In this study, we evaluated the efficacy of ensitrelvir for the treatment of cough associated with COVID-19 Omicron variants in patients with asthma. This follows the Strengthening the Reporting of Observational Studies in Epidemiology statement. A total of 223 patients were registered in this study: 121 patients chose ensitrelvir, and 102 patients chose symptomatic treatment. Cough severity, frequency, and cough-specific quality of life were evaluated using the Japanese version of the Leicester Cough Questionnaire (J-LCQ). J-LCQ documented at baseline on days 4, 7, and 14 for all patients showed a steady improvement over time in both groups. In the mixed model for repeated measures model, which accounts for repeated measurements, the change in J-LCQ score from baseline was 2.1 points higher in the ensitrelvir group (*P* <0.001). Additionally, patients who were using triple inhaled therapy at baseline showed a 2.3-point higher change in J-LCQ score from baseline (*P* <0.001). Multiple regression analysis was performed at days 4, 7, and 14, with the change in J-LCQ score from baseline as the dependent variable. Ensitrelvir was associated with scores that were 3.1 points higher on day 4, 3.5 points higher on day 7, and 2.0 points higher on day 14 compared with symptomatic treatment (*P* <0.001 for all). In conclusion, our results demonstrated that early administration of ensitrelvir may be effective as a treatment for cough due to the COVID-19 Omicron variant.

**IMPORTANCE:**

We evaluated the efficacy of ensitrelvir for the treatment of cough due to coronavirus disease 2019 (COVID-19) Omicron variant in patients with asthma. A total of 223 patients were registered in this study: 121 patients chose ensitrelvir, and 102 patients chose symptomatic treatment. Cough severity, frequency, and cough-specific quality of life were evaluated using the Japanese version of the Leicester Cough Questionnaire (J-LCQ). Multiple regression analysis was performed at days 4, 7, and 14, with the change in J-LCQ score from baseline as the dependent variable. Ensitrelvir was associated with scores that were 3.1 points higher on day 4, 3.5 points higher on day 7, and 2.0 points higher on day 14 compared with symptomatic treatment (*P* <0.001 for all). Our results demonstrated that early administration of ensitrelvir may be effective as a treatment for cough due to the COVID-19 Omicron variant.

## OBSERVATION

Chronic lung diseases are well-known risk factors for worsening the severity of coronavirus disease 2019 (COVID-19) (1, 2). However, no significant differences in hospitalization, intensive care unit admission, ventilator use, or mortality were found between a group of patients with asthma and a non-asthma group (3). On the other hand, in the post-acute COVID-19 period, patients with asthma had a significantly higher risk of cough, shortness of breath, and bronchospasm than patients without a history of asthma (4). In particular, cases with persistent cough were increased during the Omicron variant epidemic.

In Japan, three oral anti-SARS-CoV-2 drugs, ensitrelvir (Shionogi & Co., Ltd.) (5–9), molnupiravir (10), and nirmatrelvir-ritonavir (11), have been approved as therapeutic drugs against COVID-19. In addition, many people have received three or more doses of the mRNA vaccine against severe acute respiratory syndrome coronavirus 2 (SARS-CoV-2). Ensitrelvir treatment demonstrated rapid and favorable antiviral efficacy against respiratory symptoms including cough in patients with mild-to-moderate COVID-19, a majority of whom had been vaccinated (6, 7). In contrast, nirmatrelvir-ritonavir was not associated with a significantly shorter time to sustained alleviation of COVID-19 symptoms than placebo in patients who are fully vaccinated (12). Our former study demonstrated that early administration of ensitrelvir may be effective as a treatment for cough due to the COVID-19 Omicron variant (13). In addition, Yotsuyanagi et al. suggested that ensitrelvir treatment in the acute COVID-19 phase may reduce the risk of various symptoms, including cough associated with post-COVID-19 conditions (8).

In Japan, molnupiravir and nirmatrelvir-ritonavir can only be used in patients with COVID-19 risk factors for severe disease (e.g., adults 65 years of age or older, patients with malignancy, stroke, liver diseases, or immunosuppressant usage). In contrast, only ensitrelvir can be used in patients with or without COVID-19 risk factors for severe disease. A real-world database study found that ensitrelvir treatment demonstrated favorable antiviral efficacy in patients with COVID-19 risk factors for severe disease (9). In addition to cost-effectiveness, ensitrelvir is preferred over molnupiravir or nirmatrelvir-ritonavir for treating COVID-19 in Japan.

The purpose of the present study was to evaluate whether the ensitrelvir treatment can be improved for cough associated with COVID-19 Omicron variants in patients with asthma. The study was conducted in the hope that the ensitrelvir would improve the cough condition quickly compared to symptomatic treatment.

A prospective observational study was conducted in patients with asthma belonging to five facilities affiliated with the Kansai Medical University Hospital who contracted the COVID-19 Omicron variant and presented with cough between November 2022 and November 2024. This follows the Strengthening the Reporting of Observational Studies in Epidemiology statement (14). Identification of SARS-CoV-2 variants was performed as reported previously (15). The Kansai Medical University Hospital and affiliated hospitals participating in the study are deciding on a treatment plan for mild-to-moderate COVID-19 in outpatient clinics. Physicians explain the characteristics of oral anti-SARS-CoV-2 drugs and prescribe them if desired. All patients will be given symptomatic treatment and will be instructed to visit a hospital if their condition worsens.

A comparison was conducted between a group of patients who requested and took ensitrelvir (375 mg on day 1, followed by 125 mg on days 2 through 5) and a group of patients who did not request ensitrelvir and received symptomatic treatment (acetaminophen 600 mg, dextromethorphan hydrobromide hydrate 45 mg, and ambroxol hydrochloride 45 mg for 5 days). All patients in the ensitrelvir group also received symptomatic treatment. Informed consent was obtained from all patients in the form of information disclosure, and the study protocol was approved by the Ethics Committee of Kansai Medical University (approval number 2020319).

Adult patients (≥18 years) diagnosed and treated for asthma for more than 1 year and managed by pulmonary or allergy specialists in the participating hospitals were enrolled. Severity classification of COVID-19 according to the criteria of the Ministry of Health, Labour, and Welfare was as follows: mild, oxygen saturation level in room air of 96% or more, and no pneumonia shadow was observed; moderate I, oxygen saturation level in room air of 94% or 95%, and pneumonia shadow was observed; moderate II, oxygen saturation level in room air of 93% or less and a need for oxygen therapy; and severe, requirement for intensive care unit admission or mechanical ventilation. All the patients included in this study showed mild symptoms and were classified as mild severity. Patients with COVID-19 whose symptoms had been present for more than 72 hours were excluded. Patients with asthma exacerbations who require systemic corticosteroid administration or hospitalization were also excluded.

Asthma control levels were evaluated using the Global Initiative for Asthma (GINA) by physicians. Patients on medium- or high-dose inhaled corticosteroid (ICS) in combination with more than one asthma controller, i.e., long-acting β2-agonists (LABA), leukotriene receptor antagonist, long-acting muscarinic antagonist (LAMA), omalizumab, mepolizumab, benralizumab, dupilumab, tezepelumab, and systemic corticosteroid, were defined as GINA Step 4 or 5. Other patients were defined as GINA Step 1, 2, or 3. Patients were treated based on their physicians’ standard practices in a real-world clinical setting.

Cough severity, frequency, and cough-specific quality of life were evaluated using the Japanese version of the Leicester Cough Questionnaire (J-LCQ) (16–18). Cough-specific quality of life (QOL) questionnaire is more useful than the Cough Visual Analog Scale or cough score in the cough evaluation method. Thus, the American College of Chest Physicians guideline recommended the use of a cough-specific QOL questionnaire for cough evaluation of research objectives ((19)). The LCQ score is the most frequently used in the cough-specific QOL questionnaires and has been translated all over the world (17). The LCQ consists of 19 questions with a 7-point Likert scale covering three subdomains (physical, social, and psychological). Cough-specific QOL was evaluated using the J-LCQ, which was translated from the original version and validated (16). The Japanese Respiratory Society guideline for the management of cough and sputum recommends the use of the J-LCQ score for the evaluation of cough (20).

The J-LCQ scores in both groups were compared using the change from baseline as the primary outcome. First, we confirmed that there were no significant differences in most baseline patient characteristics between the patients who received ensitrelvir and those who received symptomatic treatment. The score changes from baseline were then illustrated for days 4, 7, and 14. To evaluate the overall trend, a mixed model for repeated measure (MMRM) via the “lme” command from the “nlme” package in R statistical software was conducted. After identifying significant differences between the ensitrelvir and symptomatic treatment groups using the MMRM model, multiple regression analyses were performed to assess how ensitrelvir affected J-LCQ score at each time point (days 4, 7, and 14). In both the MMRM and multiple regression analyses, binary covariates with fewer than 20 patients in total were excluded. For smoking history, current smokers and ex-smokers were combined into a single category, “smoker.” As all patients were receiving either ICS, dual inhaled therapy (ICS/LABA), or triple inhaled therapy (ICS/LABA/LAMA), ICS was used as the reference category, with dual inhaled therapy and triple inhaled therapy included as explanatory variables.

In assessing the difference in J-LCQ scores between the two groups, we should focus not only on statistical significance but also on whether the difference exceeded the minimal clinically important difference (MCID). Reportedly, the MCID of the original LCQ total score ranged from 1.3 points to 1.5 points (21). However, as the MCID for the J-LCQ score has not been established, we estimated it using the distribution method, setting Cohen’s *d* at 0.5.

**Fig 1:**
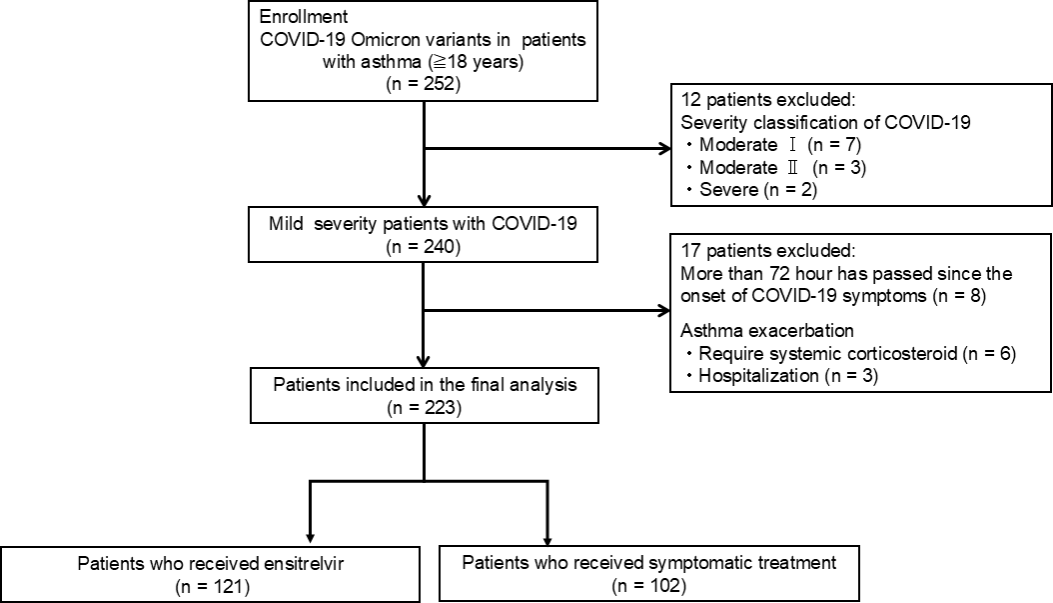
Study flow chart of the enrollment of patients.

Of the 223 patients, 121 received the ensitrelvir, and 102 received symptomatic treatment (Fig. 1). There were no significant differences in most baseline characteristics between the two groups (Table 1). However, more patients in the ensitrelvir group (43.0%) received triple inhaled therapy compared with the symptomatic treatment group (29.4%).

**TABLE 1:** Demographic and clinical characteristics of the patients at baseline^*c*^

	Ensitrelvir	Symptomatic treatment	*P*
Number	121	102	
Age	57 (45–71)	56 (44–69)	0.582
Sex, male	33 (27.3%)	28 (27.5%)	1
J-LCQ score, before COVID-19	19.4 (18.1–20.8)	19.6 (18.4–20.7)	0.639
J-LCQ score, baseline	10.5 (8.2–12.7)	10.7 (8.3–12.9)	0.582
Smoking history			0.676 (CATT)
Current smoker	5 (4.1%)	3 (2.9%)	0.730
Ex-smoker	35 (28.9%)	34 (33.3%)	0.561
Never smoker	81 (66.9%)	65 (63.7%)	0.672
Inhaled medication			0.016 (CATT)
Inhaled corticosteroids	9 (7.4%)	15 (14.7%)	0.088
Dual inhaled therapy^*a*^	60 (49.6%)	57 (55.9%)	0.419
Triple inhaled therapy^*b*^	52 (43.0%)	30 (29.4%)	0.038
Leukotriene receptor ant.	44 (36.4%)	37 (36.3%)	1
Antiallergic	42 (34.7%)	38 (37.3%)	0.779
Expectorants	13 (10.7%)	10 (9.8%)	1
Biologics	9 (7.4%)	8 (7.8%)	1
Pulmonary disease	0 (0.0%)	0 (0.0%)	1
Cardiac disease	1 (0.8%)	1 (1.0%)	1
Renal disease	3 (2.5%)	4 (3.9%)	0.705
Liver disease	4 (3.3%)	1 (1.0%)	0.379
Diabetes	17 (14.0%)	9 (8.8%)	0.296
Dyslipidemia	12 (9.9%)	9 (8.8%)	0.822
Hypertension	20 (16.5%)	11 (10.8%)	0.248
Autoimmune disorder	14 (11.6%)	16 (15.7%)	0.433
Malignancy	6 (5.0%)	1 (1.0%)	0.129
Hyperuremia	4 (3.3%)	1 (1.0%)	0.379
Cerebrovascular disease	1 (0.8%)	0 (0.0%)	1
CNS disorder	1 (0.8%)	0 (0.0%)	1
Time from onset to administration	2 (2, 3)	2 (2, 3)	0.865
No. of vaccinations	3 (2–4)	3 (2–4)	0.309
GINA treatment steps			
1	1 (0.8%)	2 (2.0%)	0.593
2	3 (2.5%)	5 (4.9%)	0.474
3	5 (4.1%)	8 (7.8%)	0.395
4	60 (49.6%)	57 (55.9%)	0.419
5	52 (43%)	30 (29.4%)	0.038
Asthma control, acute exacerbation			
0	98 (81.0%)	77 (75.4%)	0.331
1	20 (16.5%)	19 (18.6%)	0.725
≥2	3 (2.5%)	6 (5.9%)	0.306

^*a*^ Dual inhaled therapy: inhaled corticosteroids/long-acting β2-agonists.

^*b*^ Triple inhaled therapy: inhaled corticosteroids/long-acting β2-agonists/long-acting muscarinic antagonist.

^*c*^ For binary variables, the number of patients and their percentages are presented. For ordinal variables, the median and interquartile range (IQR) are shown. *P* values are provided using Fisher’s exact test for binary variables and the Mann–Whitney *U* test for ordinal variables. Additionally, for smoking history and inhaled therapy, the results of the Cochran–Armitage trend test (CATT) for 2-by-3 contingency tables are displayed.

In the combined cohort of 223 patients, the median baseline J-LCQ score was 10.5 points, with a standard deviation of 2.9 points. Using the distribution method, a change of 1.4 points in the J-LCQ score, equivalent to a Cohen’s d of 0.5, was considered the MCID. As this value is close to the previously established MCID for the original LCQ, which ranges from 1.3 to 1.5 points, we adopted 1.4 points as the MCID for the J-LCQ.

**Fig 2:**
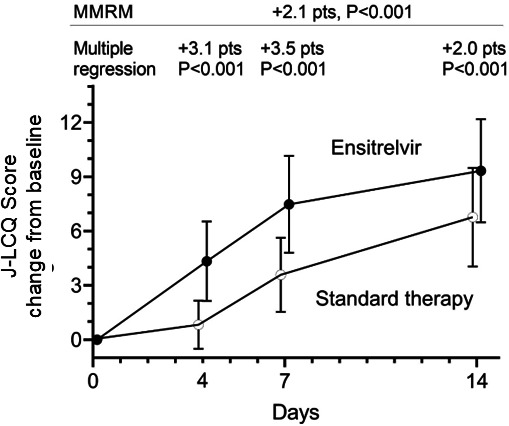
Mixed model for repeated measure analysis for J-LCQ score change from baseline.

J-LCQ documented at baseline on days 4, 7, and 14 for all patients showed a steady improvement over time in both groups (Fig. 2). In the MMRM model, which accounts for repeated measurements, the change in J-LCQ score from baseline was 2.1 points higher in the ensitrelvir group (*P* <0.001, Table 2), exceeding the MCID of 1.4 points. Additionally, patients who were using triple inhaled therapy at baseline showed a 2.3-point higher change in J-LCQ score from baseline (*P* <0.001, Table 2).

**TABLE 2:** Mixed model for repeated measure analysis for J-LCQ score change from baseline

	β	*P* value
J-LCQ change from baseline		
Day 4	2.71	<0.001
Day 7	5.68	<0.001
Day 14	8.15	<0.001
Ensitrelvir	2.10	<0.001
Age	0.02	0.015
Sex, male	0.24	0.292
Smoker (current + ex)	−0.39	0.068
Dual inhaled therapy	0.37	0.268
Triple inhaled therapy	2.27	<0.001
Leukotriene receptor antagonist	−0.46	0.09
Antiallergic	−0.13	0.632
Expectorants	−0.20	0.549
Diabetes	−0.09	0.778
Dyslipidemia	−0.86	0.019
Hypertension	0.07	0.836
Autoimmune disorder	0.00	0.995
Time from onset to administration	−0.10	0.469
No. of vaccinations	−0.15	0.06
Intercept	−2.17	<0.001

The MMRM model demonstrated the effect of ensitrelvir on the J-LCQ score through univariate analysis. However, differences in patient backgrounds between the ensitrelvir and symptomatic treatment groups are observed (Table 1). Therefore, it is necessary to confirm, through multivariate analysis, whether ensitrelvir contributed to the improvement in J-LCQ scores. Multiple regression analysis was performed at days 4, 7, and 14, with the change in J-LCQ score from baseline as the dependent variable using 15 explanatory variables. Triple inhaled therapy was one of the explanatory variables. Ensitrelvir was associated with scores that were 3.1 points higher on day 4, 3.5 points higher on day 7, and 2.0 points higher on day 14 compared with symptomatic treatment (*P* <0.001 for all, Fig. 2).

Ensitrelvir, an oral SARS-CoV-2 3Cl protease inhibitor, has shown clinical efficacy against different SARS-CoV-2 variants, including the Omicron variant (5–9). A randomized clinical trial demonstrated that 5 days of oral ensitrelvir treatment shortened the duration of cough symptoms in patients with mild-to-moderate COVID-19 (6, 7). The double-blind, phase 3, SCORPIO-SR trial assessed the effect of ensitrelvir in preventing PCC and demonstrated that relative risk reductions versus placebo were observed for some of the symptoms, including persistent cough (9). Our study demonstrated that early ensitrelvir administration may be helpful in patients with asthma.

Patients who were using triple inhaled therapy showed a higher change in J-LCQ score from baseline (Table 2). Yamaya et al. reported that LAMA reduced rhinovirus replication by reducing receptor expression and/or the number of acidic endosomes where rhinoviral RNA enters the cytoplasm (22). The same author also suggested that LAMA inhibits coronavirus replication partly by inhibiting receptor expression and/or endosomal function and that these drugs modulate infection-induced inflammation in the airway (23). In a clinical study, Holmes et al. demonstrated that LAMA is an effective treatment in nonsmoking adults with protracted cough following clinical upper respiratory tract infection (24). LAMA inhibits cough reflex sensitivity to capsaicin in subjects with acute viral upper respiratory infection (25).

In the cross-sectional, mixed-methods study including Japanese patients (≥20 years) with asthma adherent to ICS/LABA, patients with not well-controlled asthma had significantly worse cough-related health-related quality of life (HRQoL) across J-LCQ domains and significantly greater work and activity impairment versus patients with well-controlled (26). They concluded that cough burden correlated with HRQoL, suggesting that cough may be one of the key markers to inform treatment strategy for patients with asthma (26). In studies examining the frequency of asthma medication use in Japanese adult patients with asthma, the frequency of use of LAMA was very low with 4.4%–5.5% (ICS and/or LABA, 74.8%–95.7%) (27, 28). In the double-blind, randomized, parallel-group, phase 3A study (Clinical Study in Asthma Patients Receiving Triple Therapy in a Single Inhaler), in patients with uncontrolled moderate or severe asthma on ICS/LABA, adding LAMA improved lung function and Asthma Control Questionnaire-7 score (29). These findings were also confirmed in Japanese patients with uncontrolled moderate or severe asthma on ICS/LABA (30). Our present study also demonstrated that triple inhaled therapy (add LAMA to ICS/LABA) may have been effective against cough caused by the Omicron subvariant, but further research is needed.

Our study had several limitations. First, we did not evaluate viral loads such as the level of virus titers or the number of RNA copies. Further study is needed to evaluate the correlation between cough symptom and viral load. Second, the patient’s background is different from that of the general population and cannot be captured simply by the presence of asthma. Therefore, it may be difficult to conclude that the study is effective for asthma patients immediately. In addition, the MCID that we adopted in this study has not been sufficiently validated for further study. Another limitation is selection bias mainly due to the retrospective nature of this study. However, we conducted multivariate analysis to overcome this. The other limitation is the small sample size.

In conclusion, our results demonstrated that early administration of ensitrelvir may be effective as a treatment for cough due to the COVID-19 Omicron variant.

## Data Availability

The data presented in this study are available in the article.
